# It’s a long shot, but it just might work! Perspectives on the future of medicine

**DOI:** 10.1186/s12916-016-0727-y

**Published:** 2016-11-07

**Authors:** Paul Wicks, Matthew Hotopf, Vaibhav A. Narayan, Ethan Basch, James Weatherall, Muir Gray

**Affiliations:** 1 PatientsLikeMe, 10 John Street, London, WC1N 2EB UK; 2 National Institute of Health Research Biomedical Research Centre at the Maudsley, South London and Maudsley NHS Foundation Trust, London, SE5 8AZ UK; 3 Department of Psychological Medicine, King’s College London, Institute of Psychiatry, Psychology and Neuroscience, Weston Education Centre, London, SE5 9JA UK; 4 Janssen Research & Development, LLC, 1125 Trenton-Harbourton Road, Titusville, NJ 08560 USA; 5 Department of Medicine, University of North Carolina, 170 Manning Drive, Chapel Hill, North Carolina 27599 USA; 6 AstraZeneca UK, 1 Francis Crick Avenue, Cambridge Biomedical Campus, Cambridge, CB2 0AA UK; 7 Oxford University Hospitals NHS Trust, 18 Middle Way, Oxford, OX2 7LG UK

**Keywords:** Patient reported outcomes, Machine learning, Medical informatics, Smartphones, Patient engagement

## Abstract

What does the future of medicine hold? We asked six researchers to share their most ambitious and optimistic views of the future, grounded in the present but looking out a decade or more from now to consider what’s possible. They paint a picture of a connected and data-driven world in which patient value, patient feedback, and patient empowerment shape a continually learning system that ensures each patient’s experience contributes to the improved outcome of every patient like them, whether it be through clinical trials, data from consumer devices, hacking their medical devices, or defining value in thoughtful new ways.

## Healthcare on autopilot: possibilities and pitfalls for a “machine-learning” health system

Paul Wicks (Fig. 1)

**Fig. 1:**
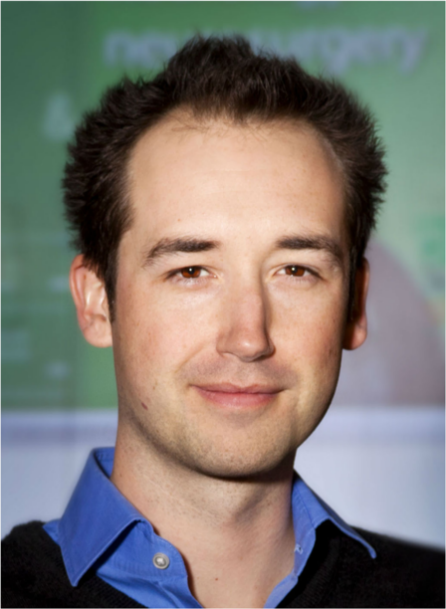
Paul Wicks is Vice President of Innovation at *PatientsLikeMe*, an online community for people living with medical conditions. Specializing in the conduct of clinical research using the Internet, Paul is responsible for shaping the scientific validity of the *PatientsLikeMe* platform and generating insights from personal health data shared by members. This sharing of online medical data has led to over 70 novel studies including a patient-led observational trial of lithium in ALS, digital tools to develop patient-reported outcome measures, a “dose-response” curve for the benefits of friendship between patients, and new methods for gaining patient input into clinical trial design. Prior to joining *PatientsLikeMe*, Paul worked at the Institute of Psychiatry (King’s College London) studying cognition and neuroimaging in rare forms of ALS, and the psychological consequences of Parkinson’s disease. In 2011, he was awarded MIT Technology Review’s TR35 “Humanitarian of the Year” award and was recognized as a TED Fellow in 2012

What was the last product you searched for online? Every aspect of the information presented to you, from its position on the screen to whether the text was underlined, was shaped by thousands of online randomized controlled experiments and scads of data being processed in real-time from millions of other customers like you [1]. Contrast this experience with the last time you received a healthcare intervention. If a treatment existed for your problem, then the (very expensive [2]) evidence for it was generated long ago from a tiny and highly unusual [3] group of volunteers who were followed up for a relatively short time period, with all the data gathered by the company with most to gain from a positive result [4], approved in a binary one-time process, and with a price shrouded in secrecy [5]. Since then, nearly all the outcome data on the success or failure of the treatment for every other person like you was either scrawled on paper in filing cabinets or, most likely, was never written down in the first place. If the outcome data was stored electronically, that was probably only for routine management or reimbursement purposes and extracting meaning from the “data shadow” is challenging [6]. Consequently, neither you nor your doctor know with confidence if the treatment they prescribed is really working for you, the manufacturer does not have much feedback as to what they could do better, and whoever paid for it does not know if they got value for money. This worries me greatly, as it seems like Amazon is leveraging every drop of data on the planet to feed my daughter the best suggestion for which episode of Peppa Pig to watch next, but that the health system has given me a paper booklet on which to record her health data.

Consumers are going to see more examples of intelligent learning systems in their daily lives. For example, Tesla cars are electric vehicles packed with sensors and a wireless transmitter that allows the manufacturer to understand how their vehicles are being used in real-time and how they cope with accidents. More recently, software updates even let software take control of the vehicle in an “autopilot” mode, the first mainstream example of a self-driving car [7]. In 2013, when an issue was identified that could cause the chassis to hit debris on the road, Tesla was able to issue an “over the air” software update rather than conduct a full physical recall – owners woke up the next morning to find their cars had been automatically updated to adjust the settings on their suspension overnight. Won’t patients expect this for their medical devices? Aren’t they right to?

In this Forum article, we look towards the future and consider how a networked medical environment has the potential to transform medicine. Hotopf and Narayan describe the potential for passive data streams from smartphones to detect signals in conditions such as epilepsy and depression and to harness novel signals such as sleep disruption or tone of voice. Basch describes how patient-reported outcomes, whether gathered in the clinic or at home through smart devices, could offer a valuable feedback loop which supplements objective passive data with actively gathered “just in time” patient reports to overlay a patient’s reported mood or feelings of aura. Weatherall highlights the opportunity for clinical trials to leap out of the clinic and on to the screen of patients’ devices to invite them to consent themselves and their data into grand distributed experiments echoing the “supercollider leap” of particle physics. Finally, Gray challenges us to think through the evolving paradigms of “value” that the future holds, and invites physicians to harness the digital data yield to minimize variation, prioritize interventions that maximize, and perhaps even fund all these endeavors by ensuring we stop paying for things that do not seem to work. In an accompanying piece from the patient perspective, Omer describes medicine at the fringes of technology and citizen science with patients taking on the mantle of “health hackers” and even “cyborgs” as they take control of their continuous glucose monitors and insulin pumps, reprogramming them and creating a community of DIY developers engaged in a process of open learning that is already outpacing the traditional medical research community [8].

Supporting these visions of the future are a number of assumptions. First, patients themselves must take control of their health [9], their data [10], and their treatment, building their own learning systems if the status quo will not satisfy their needs [11]. Today, that is only true for a subset of engaged and activated patients, mostly with chronic health conditions, and advances in behavioral science will need to disseminate the benefits to hard-to-reach populations. This will require thoughtful interaction with the built environment, transport systems, the private sector, and a tapestry of governmental departments.

Second, this amount of data, evidence, and decision-making will be unmanageable for humans to process objectively, recursively, and globally [12]. Implied therefore in the rapid, unbiased rationality and continual improvement of such systems are forms of machine learning [13], which are being tested in a range of applications that are currently small-scale, but which might expand rapidly through connected medical devices. The challenges there are that new forms of technology often undergo more scrutiny than the status quo, such as the high level of media attention surrounding the first Tesla car to crash on “autopilot mode” compared to the thousands of accidents that happen every day for cars under human control [7]. We must also address the fact that one person’s “data donation” is another person’s “invasion of privacy” [13]. Trust and transparency will be essential when a “black box” decision could affect outcomes (and share prices) on a massive scale [14].

Third, a distributed machine-learning health system must survive the attempts of entrenched interests to suppress it – while a computer system should theoretically have no conflicts of interest, prejudices, or tendency to put a positive spin on things, we can see from regulatory fines, whistleblowing lawsuits, and article retractions in medical science that a small subset of human actors will always attempt to stack the deck [15]. Even the most intelligent systems are vulnerable to “garbage in, garbage out”.

Fourth, such a system is going to have to show results quickly, which might be supported by pilots with short iteration cycles with rapid and highly visible results, such as Google Deepmind’s study of acute kidney injury [16]. By contrast, using an intelligent learning system to optimize diet and exercise to reduce heart attack risk over the course of decades will be a much less tractable problem. Other examples of quick-cycle pilots might include preventing a seizure with implanted electrodes [17], correctly classifying anomalies in medical images [18], or controlling the flow of insulin in patients wearing continuous glucose monitors [11].

Finally, the machine learning health system demands a new body of medical informatics specialists, composed of professionals as conversant with medical ethics and patient ethnography as they are with data visualization and machine code, and who will be able to protect the system from spurious data and bad actors while acting as the human oversight that permits the system to go live and begin interacting with the public. They will one day operate as “mission control” for the world’s learning health systems and their focus must always be “patients first”.

It’s a long shot, but it might just work. If one day my daughter gets sick, I hope she will be supported and cared for by a machine-learning intelligent system that knows so much more than is humanly possible.

## Remote assessment: harnessing the digital revolution for patient benefit

**Fig. 2:**
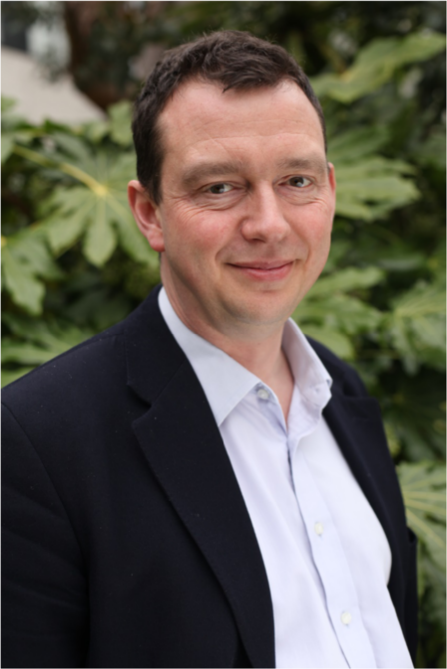
Matthew Hotopf is Director of the South London and Maudsley NHS Foundation Trust National Institute of Health Research Biomedical Research Centre (BRC) and Professor of General Hospital Psychiatry at the Institute of Psychiatry, Psychology and Neuroscience, King’s College London.He is also an NIHR Senior Investigator.. Matthew was trained in epidemiology at the London School of Hygiene and Tropical Medicine and in Psychiatry at the Maudsely. His main area of research is in the grey area between medicine and psychiatry, exploring the interaction between mental and physical health, and uses “big data” approaches to understand this interface better. He has worked extensively in areas where mental health relates to other walks of life, including occupational and military health, mental health law, and the wider community. Matthew also co-leads the Innovative Medicines Initiative RADAR-CNS (Remote Assessment of Disease And Relapse in CNS disorders) program, which seeks to use data streams from smartphones and wearables to assess and predict health states in people with epilepsy, multiple sclerosis and depression

**Fig. 3:**
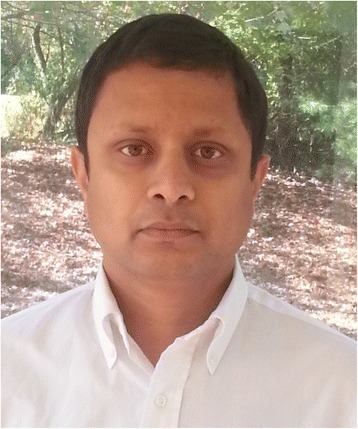
Vaibhav Narayan is Senior Director of the Neuroscience Therapeutic Area, at Janssen R&D, and Head of Neuroscience Integrated Solutions and Informatics at Janssen Neuroscience. The Neuroscience Therapeutic Area at Janssen is pioneering a more personalized and holistic approach to therapeutic intervention that goes “beyond the pill”, to offer data-driven and science-based “integrated solutions” for preventing, diagnosing, treating, and monitoring CNS diseases. Vaibhav’s work is currently focused on utilizing state-of-the-art informatics methods and digital technologies for developing markers for early diagnosis, disease progression, drug response and treatment monitoring in Alzheimer’s and mood, and to develop novel “point-of-need” tools and technologies for management of adherence and prediction of relapse in patients with Schizophrenia

Matthew Hotopf and Vaibhav A. Narayan (Fig. 2 and Fig. 3)

Changing patterns of disease within populations demand new approaches to healthcare. As life expectancy rises and mortality from cardiovascular disease and cancer falls, the proportion of the population living with other chronic and degenerative conditions has increased [19]. Amongst these, neuropsychiatric and neurodegenerative conditions place the maximum burden on societies and individuals as measured by years lived with disability, care-giver burden, and productivity loss [20, 21]. Most chronic diseases have a fluctuating course, with periods of good function interspersed with relapses and recurrences, each of which causes progressive deterioration of the underlying pathology and functional deficits. Virtually all chronic diseases share significant comorbidity with depression and other mental disorders, and these comorbidities amplify disease burden and worsen outcomes [22]. Deteriorating function, however caused, is an obvious target for secondary prevention.

Current models of disease management typically rely on patients attending secondary care providers at arbitrary intervals, with periods of more intensive care offered when the patient becomes unwell. This problem is further exacerbated as decreasing resources create pressure to see more patients in less time at increasingly infrequent intervals. Patients often feel insufficiently involved in their care, and healthcare delivery systems often disadvantage good preventive care: money is earned treating disease, not keeping people well. Further, the decisions which clinicians make are often dominated by lagging indicators of disease with little information or data to treat proactively. Systematic and reliable patient reported outcome measurement is rarely attempted and mental disorders, which contribute so much to disease burden, are widely overlooked.

In episodic disorders, such as epilepsy, clinicians rely substantially on patients’ self-report of events, often recalled over many months, to determine whether current treatments are effective or can forestall precipitous events such as seizures. A study in which intracerebral EEG sensors were used to detect epileptic activity indicated a stark difference between patients’ self-report and recorded ictal events [23]. These problems are repeated for most chronic disease. Not only is this a problem for clinical practice – where decisions about treatment choice are often made on sparse and unreliable information, but it also impacts treatment evaluation in randomized trials – where, though the process of gathering information may be more structured, the reliance on self-report adds considerable “noise” and leads to likely misclassification errors, reducing statistical power, or worse, causing misleading results due to information bias.

The digital revolution provides a potential solution to many of these problems [24]. Two thirds of the UK population now own a smartphone. Consumer wearable devices, used to monitor activity and fitness, are becoming increasingly affordable and their uptake is projected to increase exponentially across all regions of the world [25]. The sensors available from such devices provide a window into the lived experiences of patients, giving information on a patient’s symptoms, behavior and physiology via measurements of motor activity, circadian rhythms, sociability, speech, heart rate, galvanic skin response, and so on. Such passive data streams do not require the patient to do any more than use the device and consent for data to be streamed to their healthcare provider. Passively collected data can be enriched with active approaches where patients are asked about symptoms or daily stresses, or provide a platform for brief cognitive tasks, often under a challenge paradigm for increased sensitivity. Linked to suitable patient-owned health records and to the health provider’s electronic health record system, such tools would form part of an infrastructure that could increase patient engagement and participation in their own healthcare, join physical and mental health care, and provide a physician with timely and reliable information required for optimal and opportune treatment decisions.

The greatest potential for such systems may come from their ability to predict outcomes and provide targets for early intervention or prevention. For example, in recurrent depression, sleep disruption is often a sentinel symptom – an indication that a further relapse is around the corner [26]. Similarly, there is evidence that daily-life behavioral markers using mobile phone GPS and usage sensors may predict symptoms of depression [27]. In multiple sclerosis, subtle changes in symptoms, such as fatigue, or in motor function or speech prosody, may indicate an incipient deterioration in clinical state. If such signals then triggered a change in treatment which prevented relapse, the benefits could be immense. Patients would avoid relapse and there would be a strong value proposition if hospitalizations or more intense care episodes could be avoided. Healthcare systems would then move from a “diagnose and treat” to a “predict and prevent” paradigm.

This vision is at an early stage of development. Numerous building blocks need to be assembled and connected in order to understand whether this is genuinely feasible and brings the hoped-for benefits to patients and healthcare systems. First and foremost, patient involvement and trust is essential. Under what circumstances would patients be willing to share personal data? Are there certain data-streams which would be unacceptable to patients, for example, can GPS data reveal an individual’s identity? Social media, Internet shopping, supermarket reward systems, and smart ticketing systems in public transport have been widely adopted despite reasonable concerns many may have about privacy. Critical to adoption seems to be the perceived utility of digital technologies – citizens may offset concerns about privacy for the reward of better, more engaging healthcare. It remains to be seen whether uses of streamed technology in health will be perceived as too intrusive, but we anticipate that if patients own their own data and share it as they choose, many concerns of privacy may evaporate. A greater threat to the success of such technologies is probably patient fatigue – will people remain engaged, willing to wear devices over months or years? Finally, any technology which draws someone’s attention to health or risk of ill-health may cause harm. If signals are identified that predict relapse, they will need sufficient predictive value to be actionable and inevitably a proportion will be false alarms – in these circumstances, anxiety may be raised or patients be exposed to unnecessary interventions. Therefore, the adoption of such technologies – like all others – requires a careful assessment of risks and benefits specific to the use case being entertained.

A second set of challenges is the way in which data are perceived by healthcare professionals and providers. In attempting to provide tools that enhance patient autonomy and ultimately reduce unnecessary clinic visits or prevent hospitalization, remote assessment technologies may be seen as a threat – a disrupter of established practice. However, the intention should never be to replace a physician’s judgment or limit doctor-patient interactions. Instead, mobile technologies should seek to enhance clinical care, which will improve the partnership between doctors and patients. In that context, getting buy-in from the clinicians, who would be the ultimate users of patient generated and shared data, is likely to be crucial. We anticipate that due to the privileged nature of the doctor-patient relationship and the inherent trust that patients place in their doctors, the buy-in and recommendation of their doctor will be a crucial factor in a patient’s acceptance of such technologies.

Additionally, the underlying technologies, in which big data is streamed from devices, integrated with pre-existing clinical information, and rendered available in real time with nuanced interpretation, require collaboration between health and life-science sectors with expertise in the analysis of real-time streamed data, using learnings from, for example, finance and automotive industries. If these technologies are to reach the clinic and meet their potential, they will require careful evaluation in real-world settings. Such evaluation requires collaboration and input from a variety of industries and sectors, including digital, life science, academic and clinical, with strong engagement of the critical stakeholders – patients, clinical service providers, payers, and regulators. This is the vision of the Innovative Medicine Initiative’s Remote Assessment of Disease and Relapse in Central Nervous System Disorders (RADAR-CNS) project, which is a pre-competitive public private partnership co-led by Janssen and King’s College London [28]. The program will, over the next 5 years, test the acceptability, utility, and potential benefits of wearable devices and smartphone technology in multiple sclerosis, epilepsy, and depression.

## The future of patient-reported outcomes in medical research and practice

**Fig. 4:**
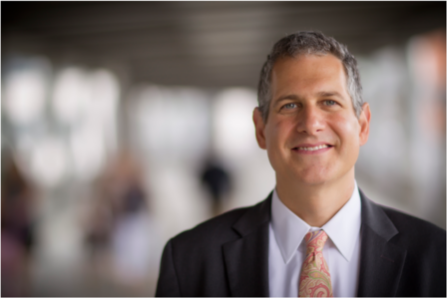
Ethan Basch, is a Professor of Medicine and Professor of Public Health at the University of North Carolina, where he directs the Cancer Outcomes Research Program. He is an oncologist and outcomes researcher whose work focuses on bringing the patient voice into clinical research and practice. Ethan’s research group has developed and evaluated multiple questionnaire and software systems for patients to report their own symptoms and side effects, including the PRO-CTCAE system for the National Cancer Institute, which is coming into use in cancer drug development trials. He currently leads two US national studies – developing patient-reported outcome quality metrics for use in oncology, and integrating PROs into routine cancer care

Ethan Basch (Fig. 4)

Patients are frequently asked to report information about their experiences in medical research and practice. For example, in clinical trials of arthritis drugs, study participants might be asked to complete serial questionnaires about their pain and mobility. During receipt of routine clinical care, patients and their caregivers might be given a survey about quality of services – asking whether they felt treated with respect or whether all of their questions were satisfactorily addressed.

Most of the time, the flow of information is unidirectional – patients report information and it goes into a black box, perhaps analyzed and used, and perhaps not. Questionnaire respondents rarely receive feedback about their own reports or aggregated results combining their information with that of other patients. Moreover, this “patient-reported outcome” (PRO) information is rarely used in real time to guide clinical care. Nevertheless, all of this is changing rapidly. PROs are being used across healthcare in novel ways to connect patients with providers, help support difficult treatment decisions, and improve management of symptoms [29, 30]; 10 or even 5 years from now, patients will likely answer questions throughout their healthcare journey via many vehicles – mobile devices, computers, phones – and interact with various automated sensors and wearable devices pouring information back to their clinical teams and to researchers. Nurses or pharmacists may receive alerts for concerning self-reports and message patients back with advice. Some of this might become automated, with guideline-recommended care advice sent electronically to patients who would benefit from it.

When facing difficult choices, the collective experiences of patients like them will be presented to predict likely outcomes. Therefore, as treatment continues, progress will be benchmarked against datasets of prior similar patients. Versions of these models are nascent but are already happening today. For example, patients considering surgery or radiation for localized prostate cancer can use predictive models to view the likelihood of various outcomes, including disease-free survival or urinary and erectile dysfunction, based on prior patients like them [31]. Then, following surgery, they can self-report their urinary and erectile functioning and benchmark themselves compared to similar patients to assess whether they are on a predicted trajectory. Their urologists see the same information, which is used as a discussion starting point regarding outcomes and symptom management.

A recent study that enabled patients to self-report symptoms during chemotherapy via the web found that nurses responded to email alerts about patients’ symptoms three-quarters of the time [32]. Most responses involved phone calls to patients to change medications and educate them about symptom control. Compared to usual care, patients using this PRO intervention experienced significantly better quality of life, fewer emergency room visits, and improved survival outcomes.

Numerous systems have been developed for collecting information from patients, and there is substantial momentum to integrate these systems into electronic health record (EHR) software platforms [29]. Several EHR vendors are creating this functionality within their own software’s patient portals, with the momentum being driven by governmental agencies interested to bring the patient voice into clinical care. Beyond enhancing care itself, there is a broad desire to make such information a standard component of EHR data to enable comparative effectiveness analyses that include PROs [33] and to evaluate quality of care based on symptom trajectories [34]. In the US, the state of Minnesota is routinely collecting PRO data for psychiatric, orthopedic, and oncologic conditions to understand comparative quality of care delivery based on how patients are feeling and functioning during or after treatment; across the UK, PROs are assessed following elective orthopedic and vascular procedures; and in Ontario, Canada, PROs are routinely collected via computer kiosks placed in oncology offices across the province [35]. Major research funding agencies have propelled this field forward, particularly in the advancement of methodology. The Patient-Centered Outcomes Research Institute (PCORI) has sponsored meetings and white papers on this topic, and the Agency for Healthcare Research and Quality has issued funding announcements specifically to evaluate the use of PROs in EHRs [36, 37]. Expert panels have been convened by the National Quality Forum and the US President’s Cancer Panel to propel this area towards standardization [38].

However, there are also major barriers. EHRs are not there yet technically; PRO functionality in EHR patient portals remains rudimentary, and most patients still do not use these portals for communication. It is challenging to collect PROs via other interfaces, such as automated telephone surveys, and then bring that information back into the EHR in an easily digestible format. This sluggish technical progress has stunted efforts to integrate PROs seamlessly with clinical and research workflow – if PROs cannot be easily collected and seen in the EHR, clinicians will not use this information, no matter how valuable it is; they simply cannot afford the extra time to use something that is not embedded within their daily system.

In conclusion, PROs are here to stay. Within the next few years, research activities will continue to assess which outcomes should be measured, what metrics to use, and the technicalities of how to incorporate PROs into existing workflows with the least inconvenience to patients and providers. Finally, the difficult task will be to determine how the information can best be used and how new models of non-real time communication, automated disease management, and decision-making can be fostered by building on information provided by engaged patients and caregivers.

## The future of clinical trials?

**Fig. 5:**
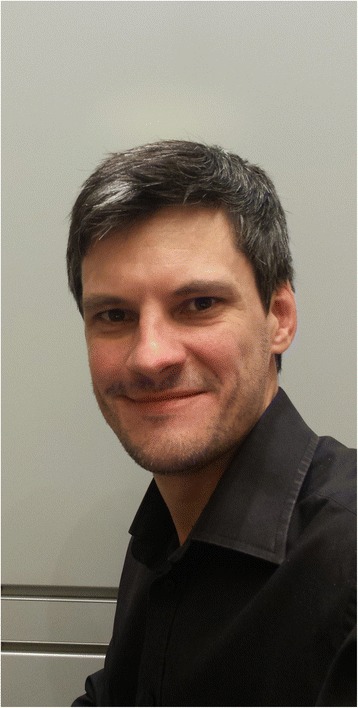
Jim Weatherall is Head of the Advanced Analytics Centre (AAC) at AstraZeneca, a department of approximately 30 clinical and health data scientists across three countries covering the disciplines of advanced statistics, scientific computing, and biomedical and health informatics. A particle physicist by training, Jim spent time as an academic researcher, before becoming a scientific software engineer consulting across a range of different industries, including the life sciences. He has had affiliate staff status at the University of Manchester since 2012, most recently being appointed as honorary reader at the University’s Health eResearch Centre. Jim and his team have introduced a number of innovations into the clinical research field, in areas such as data and text mining, data visualization, health technology evaluation, and clinical trial design

James Weatherall (Fig. 5)

There is a growing view that the clinical trials system is broken [39] or, at the very least, that it carries a high risk of not being fit for purpose in 10 years’ time. Cost and complexity are increasing exponentially, and many trials fail to recruit to target.

Other experimental paradigms have rolled with the times – for example, the evolution of the study of the most fundamental constituents of matter, particle physics. In the first half of the 20th century, key experimental findings were derived on a laboratory benchtop. The second half saw the advent of super-colliders – gargantuan apparatuses, spanning huge geographical areas, in order to respond to the ever-increasing need for higher precision and higher energy. So, what would a clinical trial look like, were it to move into the “super-collider league”?

Firstly, trialists will to have to figure out how to accommodate the plethora of new data streams that have recently come online. Personal health sensors and wearable devices offer useful ways to collect contextual per-patient data in a “stream” rather than at fixed visits, creating the promise of continuous monitoring and derivation of “digital biomarkers”. Moreover, consumer and scalable genomics is poised to be a game-changer when it comes to precision and truly personalized medicine. In the future, these and other data streams will become increasingly impossible to ignore, and data scientists will have to continually reinvent both themselves and their methods to ensure that they can make scientific sense of them.

The nature of participation in clinical trials is another rapidly moving area. “e-Patients” are becoming equals to their physicians when it comes to knowledge of disease and treatments; they are showing that they may not always wait for a conventional clinical trial to finish before analyzing their own outcomes [40]. Further, patients may use technology and the Internet to optimize their health status. Therefore, a clinical trial participant could, in parallel with their trial participation, be exchanging structured and unstructured data online with others who have the same condition. They will also be contributing a lot more self-reported information on a continuous/real-time basis following paradigms such as experience sampling rather than the filling-in of long questionnaires [41]. All of this points towards a shift in clinical trial participation from passive subjects to active research partners. What is ultimately being assessed in such trials is the combination of experimental medicine, technology, and behavioral and peer support to provide an adaptive health experience for the patient. Furthermore, there is an ever increasing drive to democratize clinical trial data and results in the public domain, and on a large scale, as can be seen through initiatives such as AllTrials and Vivli [42, 43].

The fundamental substrate of clinical trials is also changing as electronic data capture (EDC) and EHR systems move ever closer together [44]. As EHR systems mature and become more interconnected, the need for specialized EDC systems should dwindle – allowing trials to be increasingly based on data collection in routine care, with additional measurements added as required by the study at hand – the Salford Lung Study is such an example, although it is the first step on a long road to genuine "pragmatic trials" [45]. In fact, trials should become a minimally disruptive, temporary augmentation to ongoing healthcare practice. This provides unparalleled longitudinality of data, with EHRs providing data from years before to years after the study. This accumulating data could be made available in real-time to both patient and physician, as long as it does not compromise the scientific conduct of the trial. Furthermore, what if consumer technology is all that is required, foregoing the need for any standard EHR or EDC system? The asthma study launched by the Icahn School of Medicine at Mount Sinai Hospital in New York [46], based on Apple’s Research Kit [47], recruited over 4000 participants in its first 3 days [48].

So what does the clinical trial of the future look like? Here is a vision, based on a set of modular steps, each with their own challenges and opportunities:

1. The patient (potential participant) is alerted via their smart device as to clinical studies that are relevant for them. This is done automatically, via analysis of their online health record, and tuned to show the studies which would strike a balance between the best option for the patient and the most benefit to research.
2. If necessary, patients can have a discussion with their healthcare professional regarding participation to ensure that they have fully understood the implications, benefits, and risks.
3. They enroll via dynamic consent [49] on their device and receive enrolment information (if relevant) immediately.
4. Their own online health record is automatically associated with the trial’s “virtual database”, joining other participants, but without having to create a de novo data store. Their record includes their full genome sequence, enabling in-depth characterization and personalization.
5. They attend a screening visit to confirm eligibility and receive a randomization code, medications, and tech devices for continuous monitoring.
6. There are relatively few study visits after screening, with medications being regularly delivered to convenient collection locations and patients self-monitoring wherever possible.
7. Throughout the study, the patient receives regular updates on their health/disease status, as well as information about how the overall study is progressing.
8. On completion of the study, the patient receives a full report, including a personal recommendation. For instance, it could be that it would be best for them to stay on the experimental treatment as part of a long-term follow-up.

In conclusion, there are multiple directions in which clinical trials may be headed in the future, yet accurately predicting which will have gained the most traction in 10 years’ time is nigh-on impossible. Technology will come and go, as recent review studies have shown [14]; however, the best and most adaptable will survive and go on to define the future of biomedical measurement. Data scientists will understand how to make sense of heterogeneous data streams. Patients will become increasingly empowered and proactive research partners in ever more decentralized, virtualized, and flexible trial platforms. Amidst all this, the one certainty is that trials do have to roll with the times and evolve towards their own “super-collider” status.

## Education and training for value-based medicine

Muir Gray (Fig. 6)

**Fig. 6:**
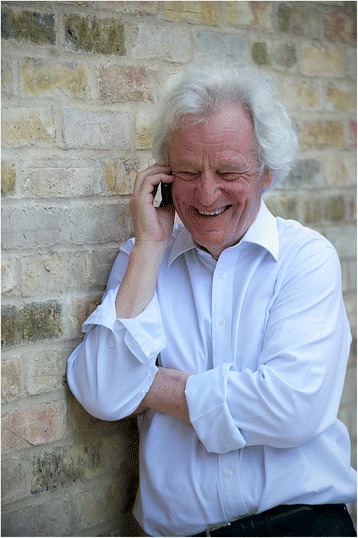
Sir Muir Gray is Consultant in Public Health at Oxford University Hospitals NHS Trust, and a Visiting Professor in Knowledge Management in the Nuffield Department of Surgery. He has been awarded both a CBE and a Knighthood for services to the NHS. Sir Gray entered the Public Health Service by joining the City of Oxford Health Department in 1972. The first phase of his professional career focused on disease prevention, and he also developed a local, then national programme of work to promote health in old age, at a time before the implications of population ageing had been recognised. Based on work in Oxford he developed a number of national initiatives, particularly designed to prevent hypothermia, publishing a Fabian Society report on the relationship between housing and poverty and the excess winter deaths, many from hypothermia, that took place in the UK. He was appointed to the board of the Anchor Housing Association and helped develop their Staying Put campaign. He has alsodeveloped all the screening programmes in the NHS, for pregnant women, children, adults and older people for example offering men aged 65 screening for abdominal aortic aneurysm and, for both men and women, screening for colorectal cancer. Working on the principle that the delivery of clean clear knowledge was analogous to the provision of clean clear water he saw the organisation and delivery of knowledge as a public health service, for example developing NHS Choices (www.nhs.uk), and setting up the Centre for Evidence Based Medicine in Oxford. During this period he was appointed as the Chief Knowledge Officer of the NHS. Sir Gray is now working with both NHS England and Public Health England to bring about a transformation of care with the aim of increasing value for both populations and individuals

The medical profession, much like all other human organizations, is constantly evolving, and it is now entering a new paradigm – the value-based paradigm. Over the past 50 years, the medical profession has played a major role in the paradigm of scientific medicine, namely medicine in which research has created remarkable opportunities in the treatment of disease –from transplantation to hip replacement. As part of this paradigm, the profession has also played a significant role in managing services and institutions to ensure that the research evidence was put into practice and that the quality of service continually improves. Progress has been highly significant, yet, three main issues remain to be dealt with by science and management.

The first of these is unwarranted variation, namely variation that cannot be explained by need or patient preference, as revealed by the NHS Atlases of Variation [50] based on the Dartmouth Atlas of Variation in the United States. These unwarranted variations in access, quality, investment, and outcome revealed two other issues: (1) overuse of lower value interventions, which always results in a waste of resources and often in unintended harm to patients, and (2) underuse of high value interventions, which always leads to failure to achieve good outcomes and often to inequity when it occurs in groups defined by age, ethnicity, or social class.

The appreciation of these problems, combined with the dramatic stimulus of the global financial collapse, has led to a new paradigm of medical practice – the value paradigm.

### The value paradigm – the doctor as creator of value for populations and individuals

When acting as a manager of resources, the doctor is responsible for the quality and safety of the service delivered to the patients who use it. Quality and safety and, increasingly, cost are the key factors in the paradigm of scientific medicine, with the doctor expected to take explicit responsibility for the management of the service in which they work, as well as being responsible for providing a good service for individual patients.

However, the three problems outlined above – unwarranted variation, overuse, and underuse – are only revealed when clinical activity is related to the population served and not just to the patients treated. The reasons for this are, partly, that there is highly variable referral to specialist services except for relatively simple health problems such as a fractured neck of femur or an acute myocardial infarction, and that, as revealed by the Dartmouth Atlas of Variation and the research related to it, cultures of clinical practice developed leading to unwarranted variation at each end of the scale, whereby clinicians operating at the higher rates and those operating at the lower rates of intervention both believe they are doing the right thing.

It is now clear that it is helpful to think of three types of value, two of which relate to the population (allocative and technical) and the third to the individual (personalized value).

- Allocative value is determined by how well the resources are allocated to the different groups within the population, aiming to reach what economists call “the point of indifference”, namely the allocation at which it would not be possible to get any more value for the population by shifting a single pound from one budget to another.
- Technical value relates outcomes to costs, the costs being considered not only in terms of money but also in terms of carbon and the time of clinicians and patients. This is a broader concept than efficiency because it takes into account not only the efficiency with which the service treats those patients that use it, but also raises issues about whether or not the patients being treated are those who would benefit most from the service in the population and whether there is over- or underuse.
- Personalized value relates the outcome to the values of the individual patient, namely the problem that was bothering them most, the value they place on a good or bad outcome, and the value they place on either taking or avoiding risks.

**Fig. 7:**
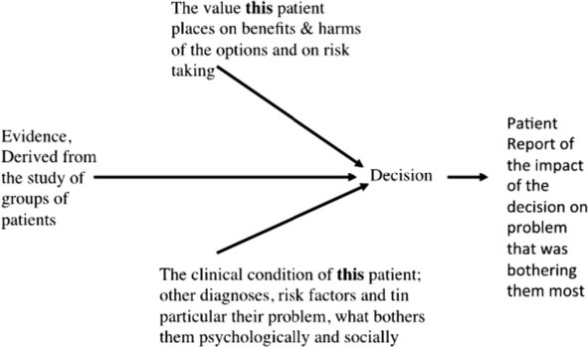
Personalization of medicine relating to a patient’s needs and values. Re-used with permission from Offox Press [51]

In the new paradigm, therefore, we are seeing the development of managerial skills as well as that of skills required for the practice of a population and personalized medicine. The term “personalized medicine” has become increasingly commonly used, with personalization relating to a patient’s needs and values (Fig. 7) [51].

The term “precision medicine” is also now used by many as a subset of personalized medicine. Personalized medicine is not a new concept, it has always been essential to make a personalized decision for an operation like a knee replacement, whereas precision medicine is a term used when genomic information is employed either in diagnosis (molecular diagnostics) or the choice of treatment (pharmacogenomics).

**Fig. 8:**
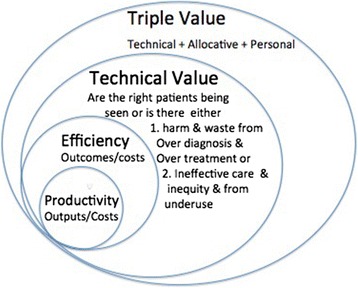
Key activities that clinicians need to develop to increase value. Re-used with permission from Offox Press [51]

Here are the five key activities that clinicians need to develop to increase value (Fig. 8) [51]:

1. Ensuring that every individual receives high personal value by providing people with full information about the risks and benefits of the intervention being offered.
2. Shifting resource from budgets where there is evidence from unwarranted variation of overuse or lower value to budgets for populations in which there is evidence of underuse and inequity.
3. Ensuring that those people in the population who will derive most value from a service reach that service.
4. Implementation of high value innovation funded by reduced spending on lower value interventions for the population.
5. Increased rates of higher value intervention, e.g., helping a higher proportion of people die well at home funded by reduced spending on lower value care in hospital in that population.

Sometimes, a new paradigm completely displaces an old one, although it may embrace and envelop it; for example, Einstein’s paradigm shift was certainly disruptive, but Newtonian physics still serves many useful purposes. The paradigm of value-based medicine embraces and envelops that of scientific medicine. It is vitally important that we continue to do research, make decisions on research-based evidence, and improve quality – this is necessary but not sufficient to meet the challenges to be faced in the decades to come. The medical profession will need to act as the stewards of the health service, as emphasized by the Academy of Medical Royal Colleges on their report “Protecting Resources, Promoting Value” [52]. The act of stewardship is to hold something in trust for the next generation and implies that clinicians cannot simply focus on what is best for their specialty. The medical profession, as a whole, must take responsibility for stewardship, and yet individual clinicians must still take responsibility for individuals; nevertheless, as it appears, this creates an difficult tension. However, a means to reconcile the two apparently segregated issues of population values and personal values seems to be emerging.

**Fig. 9:**
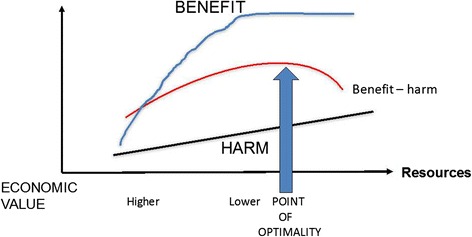
Point of optimality is reached beyond which additional resources do not cause any additional increase in value, and the reverse takes place. Re-used with permission from Offox Press [51]

The stimulus for change is arising not only from the global collapse and its consequences, but also as a result of the growing awareness of the problems of overuse and the harm that inevitably ensues. As more resources are invested in a population, the benefits may flatten but will, in fact, increase in direct proportion. Thus, as first discussed by Avedis Donabedian in 1966 [53], a point of optimality is reached beyond which additional resources do not cause any additional increase in value and, in fact, the reverse takes place (Fig. 9) [51].

**Fig. 10:**
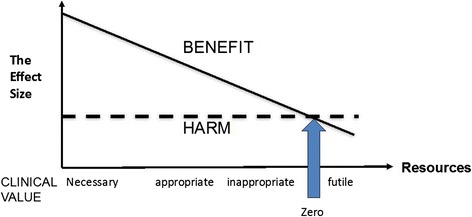
As more resources are invested in the population, the types of patients offered treatment will change, with less severely affected patients being offered treatment. The less severely affected the patient is, the smaller the benefit they will perceive, but both the probability and magnitude of harm remain constant. Re-used with permission from Offox Press [51]

As more resources are invested in the population, the types of patients offered treatment will change, with less severely affected patients being offered treatment. The less severely affected the patient is, the smaller the benefit they will perceive, but both the probability and magnitude of harm remain constant (Fig. 10) [51].

Population medicine is the new role for the medical profession. Thus, as those responsible for value, the individual clinician and the medical profession as a whole share the same goal, namely that of optimizing value.

What is also emerging with this focus on personalization and the developments in digital technology that allow the traditional functions of the doctor, such as information-giving, to be carried out digitally, is that patients highly value empathy and compassion in a clinician as well as the obvious expectations of technical excellence.
